# Self-compassion in flux: a four-year longitudinal study of intraindividual variations in self-compassion and disordered eating

**DOI:** 10.1186/s40337-026-01605-x

**Published:** 2026-04-19

**Authors:** Anders Ohnstad, KariAnne Vrabel, Yngvild Sørebø Danielsen, Per-Einar Binder, Ingrid Dundas

**Affiliations:** 1 Department of Clinical Psychology, Faculty of Psychology, University of Bergen, Årstadveien 17, 5009 Bergen, Norway; 2 Modum Bad Research Institute, Vikersund, Norway; 3 Department of Psychology, Faculty of Social Sciences, University of Oslo, Oslo, Norway

**Keywords:** Within-person, Self-compassion, Mindfulness, Disordered eating, Multilevel model, Longitudinal, Naturalistic, Self-judgment, Repeated measures, Long-term

## Abstract

**Background:**

Theoretical models suggest a dynamic interplay between self-compassion and disordered eating over time at the within-person level. Responding to periods of distress with self-kindness rather than self-judgment is assumed to reduce the risk of disordered eating, possibly by introducing self-compassion as a more effective emotion regulation strategy. Accordingly, periods of self-kindness should be associated with periods of healthier eating. However, long-term studies of the associations between moments of self-kindness and levels of disordered eating are lacking. This study aims to address this gap. We examine temporal associations between self-kindness and self-judgment and disordered eating over four years.

**Method:**

The study is a prospective longitudinal cohort study with repeated measures. A non-clinical sample of 196 individuals with an average BMI of 32.0 (SD = 6.9), who self-identified as experiencing disordered eating were examined at four time-points over a four-year period. Participants attended an eight-week low-threshold mindfulness/self-compassion - based intervention at the start of the study.

**Results:**

Within-person analyses showed that higher-than-usual (for that individual) self-kindness scores and lower-than-usual (for that individual) self-judgment scores predicted lower disordered eating at each measurement time-point.

**Conclusions:**

The results accord with findings from diary studies showing that disordered eating is less likely at times when self-compassion scores are high. A potential clinical implication is that self-compassion during times of distress (including distress caused by disordered eating), might prevent disordered eating from becoming more serious and persistent.

*Trial registration number:* ClinicalTrials.gov ID: NCT05539807, 24.08.2022

## Abstract (plain language summary)

Stress and difficult emotions may make disordered eating, such as binge eating and emotional eating, more likely. Although some people believe that harsh self-criticism is needed at these times, research indicates that self-kindness may be more effective when wanting to return to healthier eating. This study examined fluctuations in self-compassion and disordered eating of 196 individuals with self-identified eating problems at four time-points during four years. Participants attended an 8-week mindfulness-based and compassion-based intervention at the beginning of the study. The results showed that at the time points when people were kinder than usual to themselves, they were less likely to show disordered eating. Conversely, when they were more self-judging than usual, they were more likely to show disordered eating. The study is in accordance with theory and research that suggests that self-compassion training might help people cope with lapses in healthy eating patterns. Perhaps booster sessions during stressful times might prevent relapses into more serious disordered eating.

## Background

Disordered eating is prevalent worldwide [1] and can significantly impair quality of life and mental and physical health [2, 3]. Self-criticism and self-compassion have been identified as key concepts in understanding the processes associated with disordered eating [4, 5]. In a longitudinal study of initially non-symptomatic women, self-compassion protected against the onset of eating disorder symptoms [6].

Self-compassion has been conceptualized as consisting of three bipolar components: Self-Kindness vs. Self-Judgment, Mindfulness vs. Overidentification, and Common Humanity vs. Isolation [7]. Six subscales of the Self-Compassion Scale are typically used to measure self-compassion, as each pole of the three components loaded as a separate factor [7], and have been found to represent distinct but related constructs [8]. As operationalized by the Self-Compassion Scale, self-kindness means being kind to oneself when experiencing suffering and being tolerant of one’s own flaws. Self-judgment represents a tendency to be self-critical and to lack warmth, understanding, and forgiveness toward oneself when one makes mistakes, fails, or is distressed [7, 9].

Mindfulness and self-compassion-based theories and practices are integrated in several interventions [10–12] and share the notion that it is healthier to respond to one’s inner thoughts and feelings with nonjudgment and kindness, rather than avoidance and self-criticism. While mindfulness emphasizes “nonjudgmental” awareness [13], self-compassion encourages “self-kindness” which involves responding with kindness when distressing thoughts and feelings arise in awareness [7, 9–11]. Both mindfulness and self-compassion interventions have been found to represent promising approaches for managing disordered eating, particularly binge eating, emotional eating, external eating, and weight and shape concern [14–19]. In addition to mindfulness meditation and self-compassion practices, several interventions for disordered eating teach “mindful eating” [20–22]. In this approach, nonjudging, moment-to-moment awareness of mental, sensory, and bodily experiences is practiced while eating. The practice shares the characteristics of other mindfulness and self-compassion practices in being nonjudgmental and caring.

Self-compassion and mindfulness may fluctuate over time, and during periods of distress, many people treat themselves rather unkindly [23, 24]. For some, periods of life-stress and emotional distress may trigger attempts to self-soothe by over- or undereating [25, 26]. Without self-kindness and understanding, overeating episodes may in turn trigger self-judgment, shame, and self-criticism, reinforcing maladaptive eating cycles [22, 26–28]. Theories of mindfulness and self-compassion in the treatment of disordered eating suggest that, over time, increased mindfulness and self-compassion might reduce the tendency to respond to episodes of overeating (lapses) with prolonged disordered eating (relapses) [22, 25, 26]. Self-compassion may, at times of distress, replace eating with self-kindness as a more functional form of emotion regulation [29, 30]. Supporting this notion, an early experiment indicated that self-compassion could counteract a tendency among restrictive eaters to overeat after a lapse [31], and a recent review indicated that self-compassion could serve as a protective factor against the harmful effects of self-criticism [4].

The “volition stigma” might be one reason that an episode of overeating may trigger cycles of continued disordered eating in predisposed individuals [32]. This term refers to a common societal viewpoint that willpower and self-discipline are necessary to overcome disordered eating, and that people would manage to do so if they truly wanted to. Individuals affected by disordered eating may fear that responding with self-kindness to overeating episodes may inappropriately absolve them of blame and responsibility, thereby hampering their efforts to improve [32]. Another common belief is that self-kindness may mean self-indulgence [33, 34]. From this perspective, self-kindness is dangerous since it might serve as a justification for overeating [35]. For these and other reasons, some individuals may try to avoid self-kindness and self-compassion after a binge-episode. However, fearing self-compassion, for whatever reason, has been shown to be associated with greater eating disorder symptom severity rather than with less severity [27].

Mindfulness- and self-compassion-interventions typically teach participants that, even though self-indulgence may provide short-term pleasure, self-kindness involves considering what would be most beneficial in the long run. Mindful self-kindness means stopping to consider what one really needs at the moment [10, 11]. This involves mindfully directing attention to one’s needs and discerning whether one is experiencing “heart hunger” (emotional needs) and/or physical hunger, or perhaps tiredness, the need for company or time alone, or any other needs [20, 22]. According to these theories, stopping to consider (with kindness) whether one needs food and/or something else might help individuals abort cycles of overeating (such as in binge eating) and overly restrictive eating [20, 22].

### Individuals seeking low-threshold treatment

A key challenge in the field of eating disorders is the reluctance to seek professional help with eating-related challenges. It is common for individuals with disordered eating to delay help-seeking [36], and spontaneous remission is rare [37, 38]. A five-year community based study of women reporting eating disorders in the clinical and non-clinical range showed that help-seeking in any given year was only between 22% and 40% [37]. Moreover, even when individuals have sought and benefited from specialist treatment, some will need additional assistance. A rapid review reported low rates of remission and high rates of relapse for eating disorders [38]. Thus, studying individuals who do not seek specialist help, do not reach remission or experience relapses, is indicated.

### Studying self-compassion and disordered eating over long periods of time; the need for within-person studies

Within-person studies refer to what may happen to individuals over time, that is: intraindividual change [39]. Most research on disordered eating comes from between-person studies, which study differences between people (interindividual differences), and within-person studies have traditionally been less common. However, clinical work often requires an understanding of how individuals develop and cope over time [40]. Levels of disordered eating are likely to fluctuate, and the severity of disordered eating may vary depending on an individual’s emotions and circumstances at a given time. Similarly, levels of self-compassion and self-criticism fluctuate over time, since few individuals are consistently self-compassionate. Accordingly, Kelly et al. [41] propose that there is a need for a “broadening of our theoretical models and interventions to include intraindividual variability in psychological factors (states) in addition to the mean levels (traits) that differentiate individuals from each other” (p. 2). This shift in perspective may provide a more nuanced understanding of when and how certain interventions are effective, ultimately enhancing clinical and preventive efforts.

Individuals’ changes over time (intraindividual variability) may be studied longitudinally and analyzed using multilevel approaches [39, 40]. An individual’s periods of particularly high or low compassion can be identified by calculating deviations from their personal average. Such within-person studies may also examine how this variation affects disordered eating, either concurrently or prospectively. Thus, such approaches may answer questions such as: ‘When individuals experience higher-than-usual levels of self-kindness, do they also show reduced disordered eating behaviors?’

Within-person diary studies have reported that when self-compassion is high, disordered eating is less likely [42–45]. However, the question remains whether these within-person covariations persist over multiple years.

### Prior Within-person studies on the effects of intraindividual change in self-compassion

A study of non-clinical adults from the general population showed that when people were more self-compassionate than was usual for them, they experienced less stress reactivity and more positive, and less negative, affect [46]. This might also apply to individuals with disordered eating. Responding with self-compassion on stressful days could reduce the tendency to overeat as a dysfunctional coping mechanism. In agreement with this hypothesis, diary studies have reported that when self-compassion is high, disordered eating is less likely to occur [42–45]. Breines et al., [42] found this to be the case in a sample of 95 college women studied for four days, while Kelly and Stephen [44] followed a sample of 92 college women over seven days and reported similar results. Katan and Kelly [43], followed 124 women diagnosed with bulimia nervosa for two weeks, while Li et al., [45] followed 89 female Chinese employees over seven days. Collectively, these studies indicate that higher self-compassion tends to be associated with less disordered eating when both are measured at the same point in time.

### The current study

While cross-sectional and short-term longitudinal studies suggest that self-compassion co-occurs with lower disordered eating, it remains unclear whether this relationship holds over extended periods. The current study addresses this gap in the literature. The study employs a four-year longitudinal within-person design in a help-seeking, non-clinical sample. The within-person analyses enable an exploration of the dynamic interplay between self-compassion and disordered eating at an individual level over time. By moving beyond traditional between-person comparisons, the design provides insights into how fluctuations in self-kindness and self-judgment might serve as early warning signals for relapse, offering clinically actionable data that could inform and personalize treatment strategies. Since most individuals will experience lapses in their efforts to eat healthily, it is important to resume healthy eating patterns rather than enter a longer period of disordered eating, i.e. relapse. The study is also valuable because it involves a non-clinical but help-seeking sample that may be representative of individuals seeking accessible treatment without the need for referrals or diagnosis (“low-threshold” treatment). Understanding variations in self-compassion and disordered eating over longer time periods may be useful for treatment planning.

Participants in the present study were assessed at baseline, after an 8-week mindfulness-based intervention, six months later, and four years later. As indicators of self-compassion we chose two subscales from the Self-compassion Scale (SCS): Self-Kindness and Self-Judgment [7]. Since these subscales are found to represent distinct but related constructs [8], they are measured on separate scales. Our primary aim was to examine whether individual fluctuations in self-kindness and self-judgment were associated with fluctuations in disordered eating. Such patterns would suggest that when individuals treat themselves more kindly than usual, they also tend to experience less disordered eating. A secondary aim was to explore self-kindness and self-judgment as baseline predictors of disordered eating, and patterns of change at a group level.

In summary, the current study aimed to examine temporal associations between individual levels of self-kindness (and self-judgment) and disordered eating, exploring within-person variations over time. The study involved a diverse, non-clinical, help-seeking sample of individuals who self-identified as having disordered eating issues. This population is commonly encountered in low-threshold treatment settings, highlighting the study’s clinical relevance. Examining these dynamics in a naturalistic setting may provide valuable insights into how disordered eating and self-compassion covary within individuals over time.

## Method

### Pre-registration and ethical clearance

The study was approved by the Regional Committee for Medical and Health Research Ethics in Southern Norway (approval number 353216), and the study was registered in ClinicalTrials.gov ID: NCT05539807, 24.08.2022. Data for the first three registration time-points were retrospectively registered while data for the follow-up four years thereafter were prospectively registered. Data is available on reasonable request.

### Recruitment and procedure

Participants (*N* = 197) were recruited after expressing interest in a low-threshold intervention advertised on the website of the nationwide Norwegian self-help organization Rådgivning om Spiseforstyrrelser (ROS; “Advice on Disordered Eating”). The intervention offered was a group-based, low-threshold, 8-week mindfulness and self-compassion program, “Mindful Eating, Conscious Living” (MECL [20]). This intervention is now regularly conducted by the organization, but at the time it had not yet been implemented in Norway. A research project was established in collaboration with the University of Bergen and the national specialist center for eating disorders, Modum Bad. The project was later extended by four years, to explore long-term fluctuations in self-kindness, self-judgment and disordered eating. The first part of the study was made possible through funding from the Norwegian Foundation Dam, a national organization that supports health research and projects through voluntary organizations. Participants were not reimbursed for participating in the study.

Self-identified disordered eating (e.g., binge eating or emotional eating) and age ≥ 16 years were inclusion criteria. Exclusion criteria were high suicide risk, serious self-harm, active psychosis, active major depressive episode, anorexia nervosa, BMI < 18.5, and debilitating social anxiety. One participant was excluded from the analyses due to BMI < 18.5, resulting in a total sample of 196 participants (187 women and 9 men).

### Participants

The mean age of the participants was 41.0 years (SD = 12.5), and the mean BMI was 32.0 (SD = 6.9). Thirty-one participants (16%) reported height and weight corresponding to a normal BMI (18.5–24.9 kg/m²), 53 (27%) were classified as overweight (25.0–29.9 kg/m²), and 112 (57%) as having obesity (≥ 30.0 kg/m²) [47]. The mean baseline Eating Disorder Examination Questionnaire (EDE-Q) score was 3.1 (SD = 1.03). For Norwegian women, the recommended clinical cut-off score on the EDE-Q is 2.5 [48]. Table 1 presents baseline characteristics of the sample.

**Table 1:** Baseline characteristics of the sample (*N* = 196)

Variables	
Female (%) Male (%)	187 (95.4) 9 (4.6)
Mean age (SD)	41.0 (12.5)
Mean BMI (SD)	32.0 (6.9)
Normal weight (%)	31 (16)
Overweight (%)	53 (27)
Obesity (%) Reported binge eating episodes at baseline (%)	112 (57) 141 (72%)
Mean EDE-Q score at baseline (SD)	3.1 (1.02)

### Design

This was a prospective single-arm study aiming to examine associations between self-kindness and self-judgment and disordered eating over a four-year period. During this period, self-kindness and self-judgment and disordered eating were assessed at baseline (T0), immediately post-intervention (T1), six months after the intervention (T2), and four years after the intervention (T3). To minimize participant burden, the number of questionnaire items was kept low. Therefore, only the self-kindness and self-judgment subscales of the Self-Compassion Scale were included, rather than all components of self-compassion [7].

Questionnaires measuring self-kindness, self-judgment, and symptoms of disordered eating were administered by email, with a link to a secure digital survey platform, at all four assessment time-points. The study did not include a control group and therefore cannot draw conclusions regarding the effectiveness of the intervention.

### Mindful eating conscious living (MECL)

All participants took part in this 8-week group intervention with 6–10 participants at the beginning of the four-year period. As is common in mindfulness and self-compassion interventions, MECL teaches mindfulness and self-compassion more broadly, including mindful eating and non-judgmental awareness of inner experiences, and encourages self-care and self-compassion [20].

Each MECL session lasted 2.5 to 3 h and included psychoeducation, guided mindfulness practices, and group discussions. Session topics included understanding mindfulness and its application to eating-related problems; managing emotional eating; techniques for slowing down, pausing, and recognizing different types of hunger (physical versus emotional); exploring satiety and satisfaction; increasing awareness of eating behavior patterns; developing awareness of emotions and bodily sensations; examining the relationship between food and mood; and addressing cravings. The distinction between self-kindness and self-indulgence was also discussed.

Sessions were conducted face-to-face in four major cities across Norway. Trained health care workers engaged by the nationwide self-help organization facilitated the groups. Most facilitators had personal experience with disordered eating and had maintained a personal mindfulness practice for at least two years. Prior to facilitating the groups, all facilitators had completed an 11-week Mindful Eating Conscious Living (MECL) training program with the program’s originators over a two-year period.

### Measurements

#### Eating disorder examination questionnaire

The 28-item Eating Disorder Examination Questionnaire (EDE-Q [49, 50]), was used to measure severity of disordered eating. Respondents rate items on 7-point Likert scales (0–6), with higher scores reflecting greater severity or frequency of symptoms of disordered eating. Subscales are: Dietary Restraint, Weight Concern, Shape Concern, and Eating Concern. We only use the global EDE score in this study. The Norwegian translation of the scale has shown acceptable psychometric properties and Norwegian norms exist [51]. Cronbach’s Alpha of EDE-Q Global scores were 0.86 (T0), 0.92 (T1), 0.90 (T2) and 0.91 (T3).

#### Self-kindness and self-judgment

Self-Kindness and Self-Judgment were measured by two five-item subscales from the 26-item Self-Compassion Scale (SCS [7]). Respondents rate items on a 5-point Likert scale, ranging from “almost always” to “almost never.” Self-Kindness items reflect the inclination to treat oneself with kindness during moments of distress and failure, e. g. “I try to be understanding and patient towards those aspects of my personality I don’t like.” Self-Judgment items reflect the tendency to respond with criticism and judgment towards one’s own flaws and inadequacies, e.g.: “I’m disapproving and judgmental about my flaws and inadequacies. Cronbach’s Alpha of the SCS subscale Self-Kindness scores were .90 (T0), .88 (T1), .91 (T2) and .92 (T3). The Cronbach’s Alpha of the SCS subscale Self-judgment scores were .86 (T0), .92 (T1), .90 (T2) and .91 (T3).

### Missing data

A total of 31.9% of the scores were missing for EDE-Q and 33.5% of the scores were missing from the Self-Kindness and Self-Judgment scales. Data were missing at random according to Little’s MCAR-test [52] (χ² [98] = 117.26, *p* < .090). Multilevel modeling (MLM) relies on maximum likelihood estimation that allows all participants to be included in the analyses. This is a recommended approach for handling missing data, as it provides a more unbiased result than other analytic methods [53].

### Statistical analysis

In this longitudinal study, multilevel modeling was employed to address the dependency of repeated measures within a two-level design. Repeated measures were treated as data-points nested within individual patients. The session number was centered, with the first session coded as 0, serving as the intercept. Analyses were conducted using IBM SPSS Statistics (Version 27).

Intraclass correlations (ICCs) were calculated for all variables to assess variance distribution. Higher ICCs suggest greater variance *between* participants compared to *within* participants [54]. For the EDE-Q scores, 55% of the variance was attributed to between-person differences, while 45% was due to within-person variations. Self-Kindness showed 58% between-person variance and 42% within-person variance, while Self-Judgment showed 19% between-person variance and 81% within-person variance. The ICCs indicated sufficient nesting in the data to justify separating within-person and between-person effects.

To examine the between - and within-person effects, the time-varying predictors (Self-Kindness and Self-Judgment) were disaggregated, separating within-person variability from between-person variability [39, 55, 56] 

Model building began with a fixed intercept and no random effects. As the measurement points were dispersed at varying intervals of time (8 weeks from T0 to T1, 6 months from T1 to T2 and 3.5 years from T2 to T3), we tested the relative fit of using different metrics of time. We tested linear time (0, 1, 2, 3) and “real” time with 8 weeks as the smallest unit (0, 1, 3.25, 26). We also tested models that included piecewise linear time. One had the breaking point between 6-month and four years (time 1 (0, 0, 0, 1) and time 2 (1, 1, 1, 0)), while the other had a breaking point between post- and 6-month follow-up (time 3 (0, 0, 1, 1) and time 4 (1, 1, 0, 0)). Inspection of Akaike’s Information Criterion (AIC) was used to measure model fit, with a five-point decrease defined as an improvement of fit. Best fit was found for linear time (0, 1, 2, 3).

We then introduced random intercept and random slope. For all three variables (EDE-Q, Self-Kindness and Self-Judgment), a model with a random intercept and fixed slope provided the best fit. The fact that adding random slopes did not improve model fit, suggests that participants followed similar trajectories over time, making a single regression line the best representation of the data. Testing of covariance structures revealed that for EDE-Q and Self-Judgment, the Diagonal covariance structure offered the best fit, while the Identity covariance structure gave the best fit for Self-Kindness.

A series of models were then tested. Initially, time was included as the sole predictor of changes in all study variables: EDE-Q, Self-Kindness and Self-Judgment. The purpose of this was to examine change in each variable over time. To examine the dynamics of change over time, we calculated within-subjects Hedge’s g for each pairwise comparisons of means at different time-points, exploring if changes during each time span (T0 to T1, T1 to T2 and T2 to T3) were significant.

The intercept (the score at T0) was used as an indicator of the between-person score, while the individual’s deviation from their own regression line was used as an indicator of the within-person score at each time-point. For each of the two predictors, two models were tested. The first model incorporated the between-person effect, while the second model added the within-person effect. We tested Self-kindness and Self-judgment separately, providing a total of four models.

## Results

### Changes in self-kindness, self-judgment and disordered eating over time

We first investigated the nature of changes over time at a group level. The trajectories across the four measurement points demonstrated significant changes over time for all variables (Table 2).

**Table 2:** Fixed effects estimates and random effects (Variance-Covariance) estimates for disordered eating, self-kindness and self-judgment over time

Fixed effects	EDE-Q	Self-Kindness	Self-Judgment
Intercept	4.06 (0.08)***	2.49 (0.07)***	3.73 (0.06)***
Time	– 0.31 (0.03)***	0.19 (0.03)***	– 0.32 (0.03)***
Random effects			
Residual	0.42 (0.78)–0.97 (0.20)	0.38 (0.03)	1.84 (0.34) -0.27 (0.49)
Intercept	0.74 (0.74)	0.53 (0.07)	0.38 (0.06)
Akaike	1544.39	1278.11	1310.09

EDE-Q: Eating Disorder Examination Questionnaire. *p < .05, **p < .01, ***p < .001

EDE-Q (*B* = – 0.31, *SE* = 0.03, *t* (232.30) = – 8.95, *p* < .001) and Self-Judgment (*B* = – 0.32, *SE* = 0.03, *t*(245.67) = – 9.57, *p* < .001) decreased over time, while Self-Kindness increased over time (*B* = 0.19, *SE* = 0.03, *t*(362.50) = 6.81, *p* < .001). A dependent-samples Hedge’s g was calculated as an effect size measure (Table 3).

**Table 3:** Magnitude of change over time

Time intervall	EDE-Q Hedge’s g [95% CI]	Self-Judgment Hedge’s g [95% CI]	Self-Kindness Hedge’s g [95%]
T0 to T1	– 0.52 [– 0.65, – 0.40]*	– 0.60 [– 0.73, – 0.46]*	0.50 [0.37, 0.64]*
T1 to T2	– 0.09 [– 0.22, 0.04]	0.06 [– 0.07, 0.20]	0.00 [– 0.14, 0.14]
T2 to T3	0.01 [– 0.17, 0.18]	0.02 [– 0.15, 0.19]	– 0.06 [– 0.21, 0.09]

* The 95% confidence interval does not include zero, indicating a reliable effect; T0 = Baseline, T1 = after the 8-week intervention, T2 = 6-months later, T3 = four years later.

When comparing pre-intervention (T0) with post-intervention scores (T1), there was a moderate effect, indicating that there had been a favorable change during the 8 weeks of the intervention. No further changes were observed from post-intervention (T1) to the 6-month follow-up (T2) or from the 6-month (T2) to the four-year follow-up (T3), indicating that the changes might have been sustained.

### Self-kindness

A summary of fixed and random factors for Self-Kindness as a predictor of disordered eating is shown in Table 4.

**Table 4:** Between- and within-person effects of self-kindness on disordered eating

Predictor: self-kindness		
Parameter	Model 1	Model 2
Fixed effects		
Intercept	5.88 (0.25)***	5.64 (0.26)***
Time	– 0.31 (0.03)***	– 0.30 (0.03)***
Between-effect	– 0.73 (0.10)***	– 0.64 (0.10)***
Within-effect		– 0.38 (0.07)***
Random effects		
Residual	0.42 (0.07)–0.93 (0.19)	0.39 (0.07)–0.75 (0.16)
Intercept	0.52 (0.08)	0.53 (0.08)
Akaike	1498.25	1429.87

Residuals are given as a range across the four time points

*p < .05, **p < .01, ***p < .001.

In Model 1, the between-persons effect was significant (*B = *– 0.73, *SE* = 0.10, *t*(181.47) = – 7.50, *p <* .001) indicating that the between-person variance in baseline Self-Kindness influenced the level of disordered eating across the four time points. In the second model, both the between-person effect (*B = *– 0.64, *SE =* 0.10, *t*(194.11) *=* – 6.44, *p <* .001) and the concurrent within-persons effect (*B =* – 0.38, *SE =* 0.07, *t*(312.57) *=* – 5.79, *p <* .001) were significant. This implies that deviation from an individual’s expected level of Self-Kindness on a particular time point predicted their concurrent level of disordered eating.

#### Self-Judgment

These sequences of testing two models were repeated for Self-Judgment as a predictor. Table 5 shows a summary of fixed and random factors.

**Table 5:** Between- and Within-Person Effects of Self-Judgment on Disordered Eating

Predictor: Self-Judgment			
Parameter	Model 1		Model 2
Fixed effects			
Intercept	– 3.29 (0.93)***		– 1.17 (0.98)***
Time	– 0.32 (0.03)***		– 0.35 (0.04)***
Between-effect	1.97 (0.25)***		1.40 (0.26)***
Within-effect			0.36 (0.06)***
Random effects			
Residual	0.42 (0.07)–1.03 (0.20)		0.39 (0.07)–1.85 (0.40)
Intercept	0.51 (0.08)		0.49 (0.73)
Akaike	1491.45		1427.79

Residuals are given as range across the 4 time points. *p < .05, **p < .01, ***p < .001

In model 1 the between-persons effect of Self-Judgment was examined with respect to its effect on disordered eating (EDE-Q) as a dependent variable. The between-persons effect of Self-Judgment on disordered eating in model 1 was significant (*B =* 1.97, *SE =* 0.25, *t*(178.80) *=* 7.90, *p* < .001). This indicates that individuals with higher levels of self-judgment when entering the intervention tended to have higher levels of disordered eating over time across the four years of the study. In model 2 the concurrent within-persons effect of Self-Judgment on disordered eating (EDE-Q) was added. The between-persons effect of Self-Judgment (*B =* 1.40, *SE =* 0.26, *t*(234.95) *=* 5.33, *p* < .001) was significant, as well as the concurrent within-persons effect (*B =* 0.36, *SE =* 0.06, *t*(333.15) *=* 6.13, *p* < .001). This implies that a participant’s deviation from his or her expected level of self-judgment at a particular time point predicted his or her concurrent level of disordered eating.

## Discussion

This long-term study examined within-person associations between self-kindness, self-judgment, and disordered eating over a four-year period in 196 non-clinical but help-seeking participants with self-identified disordered eating. Overall, the findings suggested that fluctuations in self-kindness and self-judgment were associated with concurrent fluctuations in disordered eating. Periods of higher-than-usual self-kindness were associated with lower levels of disordered eating, whereas periods of higher-than-usual self-judgment were associated with higher levels of disordered eating.

Preliminary ICC analysis suggested that there was sizable within-person variability in all variables during the study. Almost half (45%) of the variance in disordered eating during the study was due to variations at the within-person level, suggesting that disordered eating will fluctuate in samples such as the present. Similar within-person variability was found for self-kindness (42%). There was remarkably high within-person variability in self-judgment (81%), indicating that it was normal in this sample to experience low levels of self-judgment at times and higher self-judgment at other times.

Within-person variability in disordered eating has also been found in other studies. For example, within-person variability in disordered eating was 23% in a four-day study in a non-clinical sample [42], 31% during a 12 week study in a clinical sample [40] and 77% during a 13-week study in another clinical sample [57]. Within-person variability over time for self-compassion in these three studies varied from 37% to 66%. This supports the assumption that individuals may vary in their levels of disordered eating, self-kindness and self-judgment over time.

The secondary aim of this study was to explore baseline predictors of disordered eating and group-level patterns of change across the study period. Individuals who entered the study with higher self-kindness and lower self-judgment showed less disordered eating, indicating that these characteristics might represent protective factors. This finding is consistent with prior research showing that self-compassion is typically associated with lower levels of eating pathology in between-person studies [5, 58]. Analyses of group level changes during the four years of the study showed that self-judgment and disordered eating decreased while self-compassion increased during the intervention, with no additional significant changes subsequently. Although it cannot be determined whether these changes were due to the intervention, similar long-term patterns have been reported elsewhere. For example, a study of university students reported sustained benefits for up to six years after participants had completed a seven-week MBSR course with booster sessions [59]. This suggests that changes after a short mindfulness-based intervention may be sustainable. Due to the design of the present study, no conclusions can, of course, be drawn regarding the effectiveness of the intervention from this or other uncontrolled studies. Further studies are needed to examine the replicability and meanings of the observed patterns of change.

Summarizing, the results support diary-studies that show that disordered eating co-varies with fluctuations in self-compassion [42–45]. However, the finding that these associations persist over periods of several years has not, to our knowledge, been reported previously. The consistent relationship between self-compassion and disordered eating across extended timeframes indicates that self-compassion is indeed implicated in understanding individual trajectories of disordered eating. This is in accordance with theory and research suggesting that self-compassion may be important for understanding and treating disordered eating (e.g. Turk and Waller [5]). Showing that self-kindness and self-judgment and disordered eating fluctuate together in individual trajectories measured over several years brings the field one step closer to understanding the mechanisms that may be involved in disordered eating.

### Clinical implications

The findings suggest that assessing clients’ ways of treating themselves when distressed might help therapists evaluate when clients’ individual relapse risk is high. Prior studies suggest that when distress is accompanied by self-judgment, self-criticism and shame, overeating may result as a maladaptive coping response [42–45]. Building on these results, therapists might use periods of client self-judgment as a warning signal, and periods of greater self-kindness as an indication of movement towards improvement [40]. They might explore with clients their individual triggers of self-judgment and introduce alternative ways of coping with distress [10, 60]. Given these insights, clinicians may tailor follow-up and relapse prevention programs to individual clients in their unique interpersonal contexts. For example, some clients may find themselves exposed to societal pressures to change their body weight, with others telling them that being self-compassionate will hamper their efforts to change their disordered eating [32]. If clinicians explore at which times and under which circumstances their clients face challenging situations, they can help their clients monitor their state of self-compassion at those times.

Individuals not currently in treatment might benefit from monitoring their self-compassion levels and seeking help when they find themselves responding to setbacks with only self-judgement. This self-awareness, combined with compassionate support from others, might help prevent brief lapses from developing into more serious relapses. Thus, the results may be useful for individuals experiencing disordered eating, regardless of whether they currently receive professional help or not. Being mindful of periods when they have lost their ability to recover from prolonged self-judgment and shame, might help them recognize when additional support, such as a booster self-compassion session, could be beneficial [10, 22, 61].

### Strengths and Limitations

Longitudinal studies offer insights into changes over time. They come, however, with challenges, particularly when involving extended follow-up periods. A strength of longitudinal multilevel analyses is the inclusion of all available data regardless of attrition. This method can accommodate participants with varying numbers of observations and enables the disaggregation of within- and between-person change and allows researchers to identify individual trajectories over time.

Still, statistical analyses cannot fully compensate for the high attrition that was observed in the present study. Reimbursing participants for completing study assessments might have been helpful, but may raise ethical concerns [62]. Furthermore, the unequal and sparse spacing of measurement time points is a limitation [63]. More frequent assessments would have allowed for a more stable and representative estimate of the associations between self-kindness, self-judgment, and disordered eating.

We focused on concurrent within-person associations between self-kindness, self-judgment and disordered eating. It could be argued that a lagged within-person model would be better suited to understand the causal relationship between self-compassion and disordered eating. Lagged within-person models were not chosen for two design reasons. First, the assessment intervals were highly unequal, making a single discrete-time lag parameter difficult to interpret. Second, with only four waves (three potential lag intervals per participant), lagged estimates are potentially unstable, particularly in the presence of large mean shifts around treatment. By contrast, concurrent within-person associations can be estimated with fewer assumptions and directly address whether improvements in self-compassion co-occur with reductions in disordered eating at the same assessment.

To what extent these results generalize is not known. It may be that mindfulness and self-compassion-based interventions are particularly suited for nonclinical samples with a preponderance of binge eating, perhaps because patients in a clinical setting may be more fearful of self-compassion than nonclinical samples [27]. Due to the lack of formal diagnosis in the present study, more research is needed to examine the types of disordered eating to which these results may apply. Finally, this study did not register all aspects of self-compassion that have been shown to correlate with eating [64]. Nor were additional salient variables such as shame and fear of self-compassion examined. Further studies are needed to examine the role of these variables over time.

## Conclusion

The study showed that disordered eating was more likely to occur during periods of low self-compassion. Being able to face periods of stress and emotional distress with self-kindness rather than (only) self-judgment seems to be essential. This ability might help individuals to remain on a trajectory of healthy eating, experiencing only short intermittent lapses in their efforts to eat healthily, rather than enduring relapses to disordered eating.

## Data Availability

Data are available upon reasonable request.

## Abbreviations

EDE-Q: Eating Disorder Examination Questionnaire
ICCs: Intraclass correlations
NGO: Non-Governmental Organization
SCS: Self-Compassion Scale
